# Design and Implementation of High-Capacity DDR3 Micro-Module Based on 3D TSV Advanced Packaging

**DOI:** 10.3390/mi17040459

**Published:** 2026-04-09

**Authors:** Haoyue Ji, Liang Zeng, Hongwen Qian, Wenchao Tian, Jingjing Lin, Yuhe Duan

**Affiliations:** 1 School of Electro-Mechnical Engineering, Xidian University, Xi’an 710071, China; wctian@xidian.edu.cn; 2 The 58th Research Institute of China Electronics Technology Group Corporation, Wuxi 214062, China; 15970829277@163.com (L.Z.); hongwen_qian@sina.com (H.Q.); linjingjing112@163.com (J.L.); 13921533133@163.com (Y.D.)

**Keywords:** multi-level collaborative design, wafer-level packaging, wafer-level fan-out, 3D TSV

## Abstract

To meet the demands for miniaturization, lightweight design, and high performance in modern electronic systems, advanced 3D TSV technology enables a substantial increase in storage capacity even within physically constrained form factors. This paper proposes a schematic design methodology and system-level integrated modeling approach for a four-layer stacked micro-module based on wafer-level packaging. By leveraging heterogeneous chip fan-out technology and TSV-based vertical stacking, the fabricated DDR3 micro-module achieves a compact footprint of 14 × 9 × 3.5 mm, a storage capacity of 4 GB, and a 64-bit bus width. Compared to conventional board-level mounting, the module reduces the footprint area by 95%. Following comprehensive multi-level testing, the micro-module fully complies with standard protocol requirements, enabling a paradigm shift in form factors for mobile computing devices while enhancing computational density and energy efficiency in data center server applications.

## 1. Introduction

Driven by advancements in science and technology, as well as growing market demands, electronic products such as smartphones, tablets, and drones impose extremely stringent requirements on spatial dimensions and performance parameters [1,2]. As a high-performance memory component, DDR3 is widely used in computing memory systems, embedded systems, and industrial and automotive applications, where it serves the function of temporary data storage to support high-load and multitasking operations.

Conventionally, the approach of discretely arranging a single DDR3 chip along with several decoupling capacitors and terminating resistors on the motherboard consumes a significant amount of PCB area [3,4]. In addition, long board-level traces can lead to signal reflection, crosstalk, and attenuation, thereby limiting the maximum stable operating frequency of the system [5].

The adoption of stacked packaging technology based on wafer-level packaging enables the integration of multiple DDR3 chips into a single package. On one hand, this can effectively reduce the occupied motherboard area, achieving the goals of miniaturization and weight reduction. On the other hand, the micro-module can shorten the interconnection distance with processors (such as CPU/FPGA) and optimize internal traces, which reduces signal transmission loss and delay [6]. This allows the system to operate more stably at a higher frequency and improves the overall performance margin of the system [7].

To meet the stringent demands of modern electronic devices in terms of integration density, performance, and the size factor, this paper proposes a design methodology and process implementation scheme for a high-capacity DDR3 memory device based on advanced 3D packaging technology. In this scheme, 2.5D packaging technology is first employed to integrate the memory chip and TSV interposer chip into a single-layer fan-out micro-module through a wafer-level fan-out process. Four such single-layer components are then vertically stacked to achieve 3D packaging integration [8,9]. The entire process flow does not rely on restricted equipment such as EUV lithography, forming a differentiated development path compared to HBM technology routes that depend on advanced overseas processes, thereby offering greater self-reliance and technological autonomy [10,11]. At the design level, a multi-level co-design workflow was established, enabling joint simulation and parameter optimization across the chip, package, and substrate. This effectively resolves the iteration issues commonly encountered in traditional serial design workflows due to separation between design levels [12]. At the process level, focusing on warpage control as a critical bottleneck in multi-layer stacking, systematic warpage simulation and process parameter optimization were carried out to provide a process window for high-yield, high-reliability engineering applications [13].

## 2. Design Method for Multi-Layer Stacked High-Capacity DDR3 Memory Micro-Module

### 2.1. Schematic Design of Multi-Layer Stacked DDR3 Micro-Module

With the increasing demand for memory bandwidth and capacity in electronic systems, chip-level stacking has become a common approach for System-in-Package (SiP) memory integration. For DDR3 micro-modules, two main three-dimensional integration methods are typically employed: through-silicon vias (TSVs) and wire bonding (WB). Both techniques enable miniaturization and high integration density [14,15]. Based on actual application requirements, this work utilizes a DDR3 die with a storage capacity of 1 GB and a bit width of 16 bits and achieves the creation of a DDR3 micro-module through four-layer three-dimensional stacking. Since capacity scales linearly with the number of stacked layers, the resulting DDR3 micro-module offers a total capacity of 4 GB and a 64-bit bus width.

**Figure 1:**
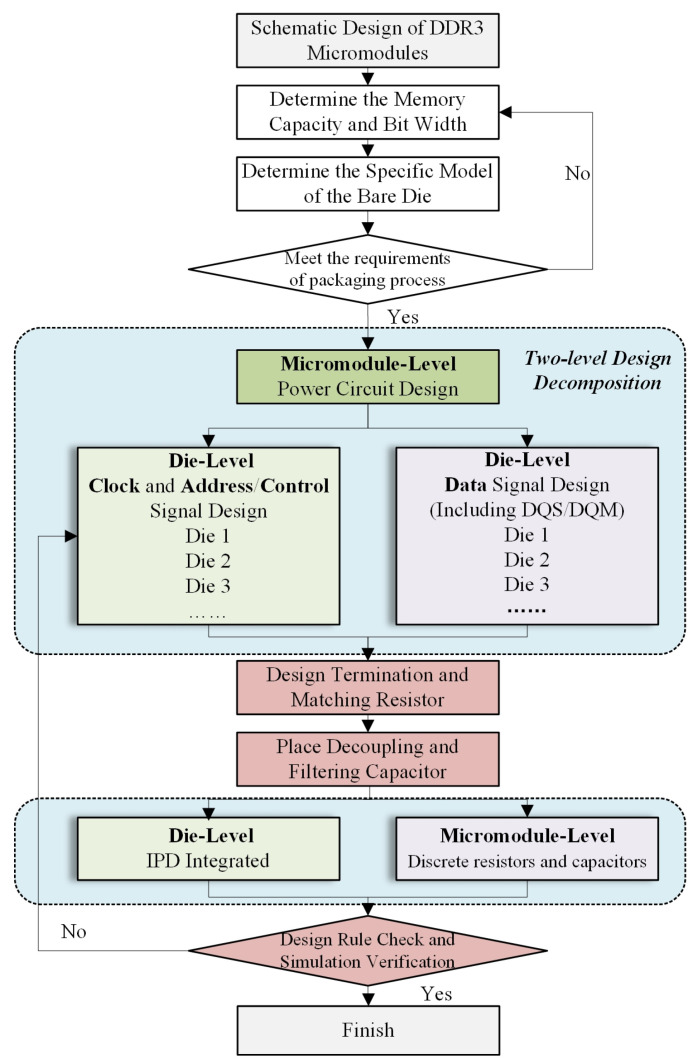
Schematic design process for DDR3 micro-module based on multi-layer stacking.

After determining the chip selection and the number of stacking layers, the careful planning of the power supply circuits and signal groups within the micro-module is required. The design of the micro-module can be divided into two levels: the die level and the micro-module level. The overall design flow is illustrated in Figure 1.

In the micro-module-level design, it is essential to complete the core power supply design for V_DD_ and V_DDQ_. Adequate decoupling capacitors should be arranged, or alternatively, an integrated passive device (IPD) can be adopted [16]. For the V_REF_ power supply, a dedicated low-dropout regulator (LDO) is required, along with appropriate filter capacitors. Since the proposed micro-module integrates four stacked DDR3 chips, which results in a large capacitive load and insufficient pin driving capability, it is necessary to incorporate a V_TT_ termination pull-up power supply.

**Figure 2:**
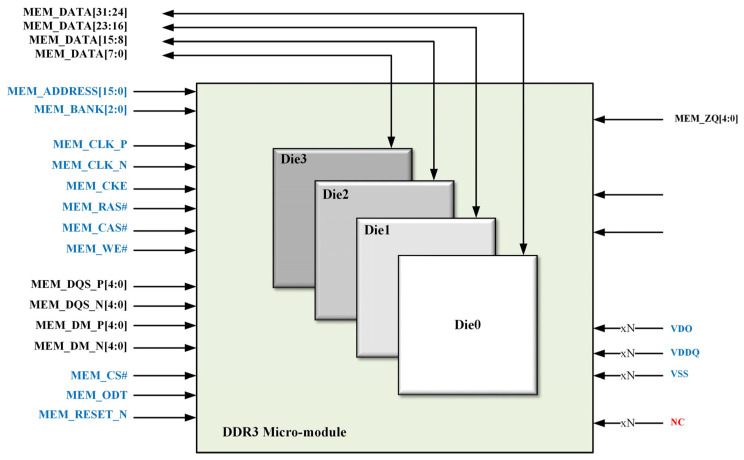
Schematic Diagram for DDR3 Micro-module Based on Multi-Layer Stacked.

In terms of signal design, considering the multi-chip integration characteristics of the micro-module, the applicable design topology for the multi-chip stacked micro-module must be analyzed from the perspectives of multi-load circuit theory, channel impedance, and routing design (e.g., trace delay). For chip-level routing, a design strategy emphasizing equal-length and short connections is adopted for clock signals (CLK/CLK#), address/control signals, and data signal groups. A schematic diagram of the four-layer stacked micro-module is shown in Figure 2.

### 2.2. Signal Transmission Simulation of Multi-Layer Stacked DDR3 Micro-Module

DDR3 SDRAM achieves high-speed data transmission by transferring data on both the rising and falling edges of the clock signal. To meet the electrical characteristics and specifications of the DDR3 interface, it is essential to consider timing requirements and signal integrity issues such as reflection, crosstalk, and power supply noise [17,18]. Therefore, simulation is necessary to verify whether the design can operate at a target frequency and maintain data integrity across different operating conditions.

**Figure 3:**
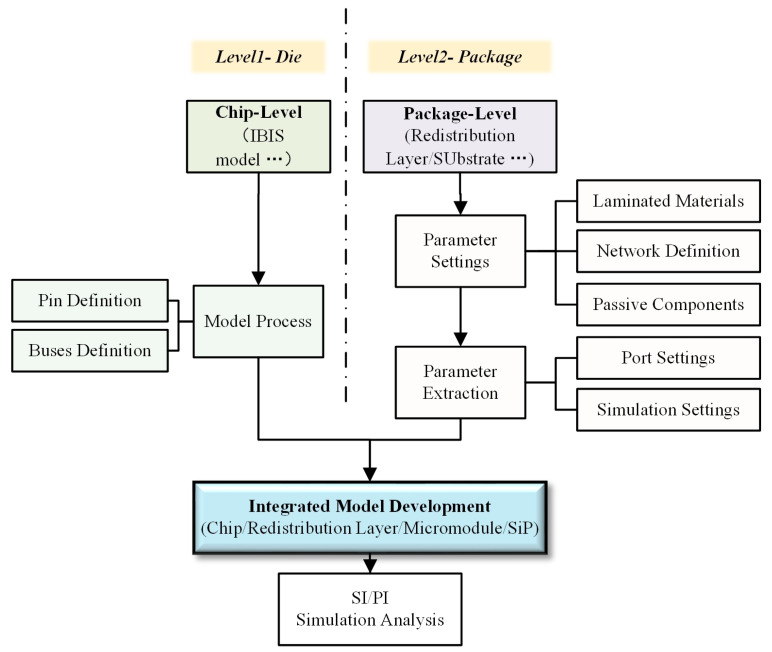
Collective modeling method for multi-level cascade model of multi-layer DDR3 micro-module based on wafer-level packaging.

The multi-layer stacked DDR3 micro-module consists of various physical components, including chips, single-chip redistribution layers (RDLs), and through-silicon vias (TSVs). Accordingly, a bottom-up modeling approach is adopted to establish a multi-level cascaded simulation model for the “chip (including RDLs)–micro-module” system. This model is designed to characterize the electrical behavior along the complete transmission path—from the interior of the chip, through the TSVs of the wafer-level package (WLP), and into the redistribution layers. The overall modeling methodology for the multi-level cascaded model of the wafer-level packaged multi-layer DDR3 micro-module is illustrated in Figure 3.

**Figure 4:**
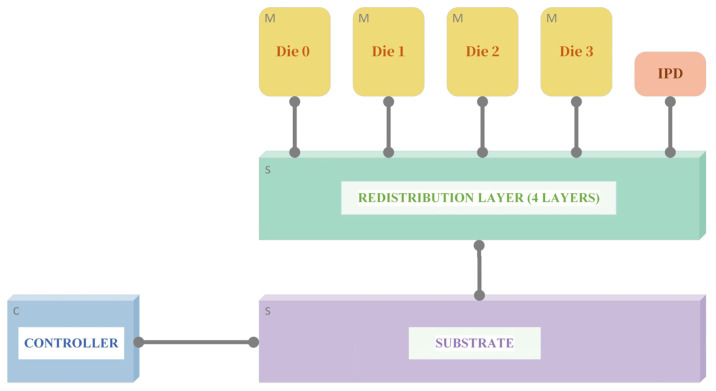
Cascaded simulation topology of DDR3 micro-module.

After establishing the integrated cascaded model, signal integrity simulations are performed on the DDR3 micro-module. These simulations include the construction of a Fly-by topology, time-domain and frequency-domain analyses, and evaluations of waveforms, eye diagrams, and timing parameters. Signal timing optimization is achieved by considering factors such as transmission delay, rise/fall time, skew, and jitter, with adjustments made to signal delay, driver impedance, crosstalk mitigation, and power supply noise reduction [19]. The detailed signal integrity simulation settings are listed in Table 1, and the simulation topology—encompassing the chip, redistribution layer, and substrate—is illustrated in Figure 4.

**Figure 5:**
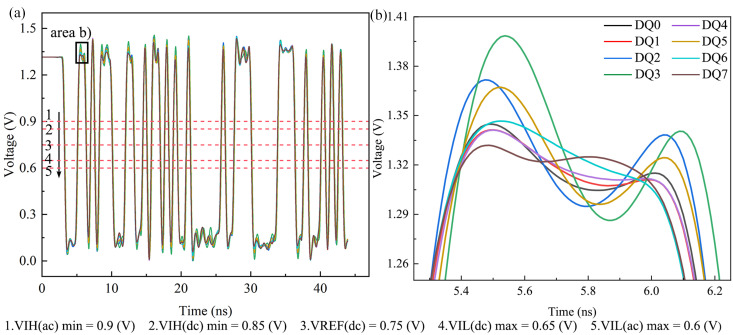
(**a**,**b**) Signal waveform diagrams of DQ lines (DQ0–DQ7) in memory.

**Figure 6:**
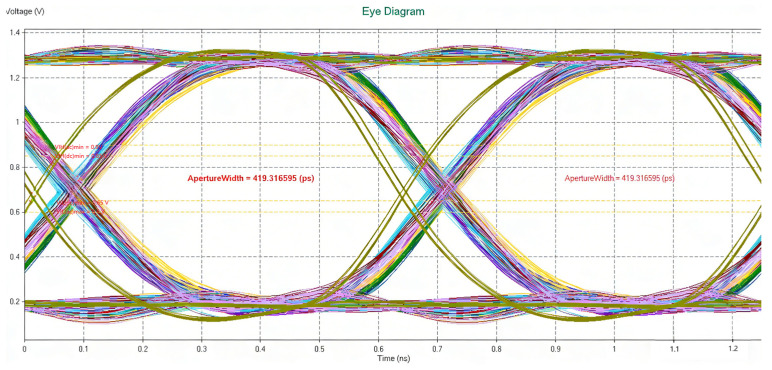
Eye diagrams of DQ lines (DQ0–DQ7) in memory.

The following conclusions are drawn from the simulation analysis:

- Address signals: With the termination pull-up resistance set to 39.2 Ω and pulled up to 0.75 V_TT_, all address signal waveforms satisfy the judgment criteria under both random and identical patterns. The simulations performed using the software’s default stimulus delay configuration (Add-0T, CLK-0.5T) show that the timing between address signals and the clock complies with the JEDEC JESD79-3 protocol.
- Control signals: Under the same termination configuration (39.2 Ω pull-up to 0.75 V_TT_), all control signal waveforms meet the judgment criteria for both random and identical patterns. With the default stimulus delay settings (Ctrl-0T, CLK-0.5T), the timing between control signals and the clock also satisfies the protocol requirements.
- Clock signals: The clock signals meet the specified requirements.
- Data signals:
  - Data writing: Using the same random pattern, all data write signals satisfy the timing requirements. When different random patterns are used to simulate the timing between data signals (DQ) and strobe signals (DQS), the write signals continue to meet the timing requirements. Additionally, the timing between the strobe signals (DQS) and the clock complies with the protocol.
  - Data reading: Using the same random pattern, all data read signals satisfy the timing requirements. The waveforms and eye diagrams of the DQ0–7 signals are shown in Figure 5 and Figure 6.

Signal integrity simulations were performed at a data rate of 1600 Mbps. The results show that the DQ channels exhibit no significant waveform distortion, the eye diagrams are well-opened, and both eye height and eye width provide sufficient margins. The observed waveform differences among the DQ channels are primarily attributed to variations in trace length and parasitic parameters along RDL, TSV, and micro-bump paths. All simulated parameters meet the JEDEC standard requirements, thereby validating the signal transmission reliability of the proposed micro-module at the target data rate from a simulation perspective.

**Figure 7:**
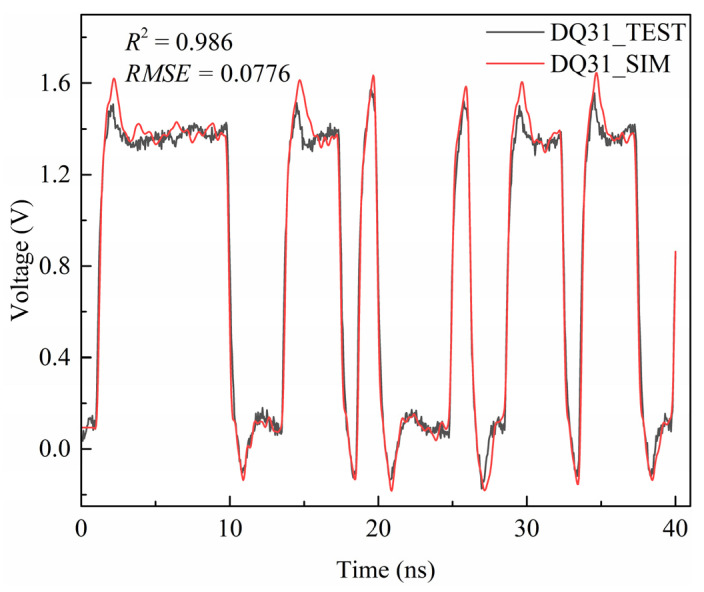
Comparison between simulated and measured results of DQ31 line.

To further verify simulation accuracy, signal testing was conducted, and DQ31 was selected for comparison. A comparison between the simulated and measured results is presented in Figure 7. A total of 999 voltage data points were collected within the time range of 0 to 40 ns. A statistical analysis of these 999 data points yielded a coefficient of determination (R^2^) of 0.986 and a root mean square error (RMSE) of 0.0776 V between the simulated and measured values. These results demonstrate strong consistency between the established simulation model and the experimental measurements, with the prediction error controlled within 8% of the signal swing. This confirms the accuracy and engineering practicality of the multi-level cascaded simulation method proposed in this work.

## 3. Process Design for Multi-Layer Stacked High-Capacity DDR3 SDRAM

To enable efficient integration and shorten the research and development cycle for micro-system products, this paper presented the design of a four-layer stacked DDR3 micro-module based on wafer-level fan-out packaging technology [21]. The core process involves heterogeneous integration between functional chips and prefabricated TSV interposers through wafer reconstruction, enabling the precise I/O routing of DDR memory chips and connection to TSVs [22]. On this basis, high-density solder bumps are employed to realize three-dimensional vertical electrical interconnections between multiple chip layers, resulting in a high-capacity DDR3 micro-module.

**Figure 8:**
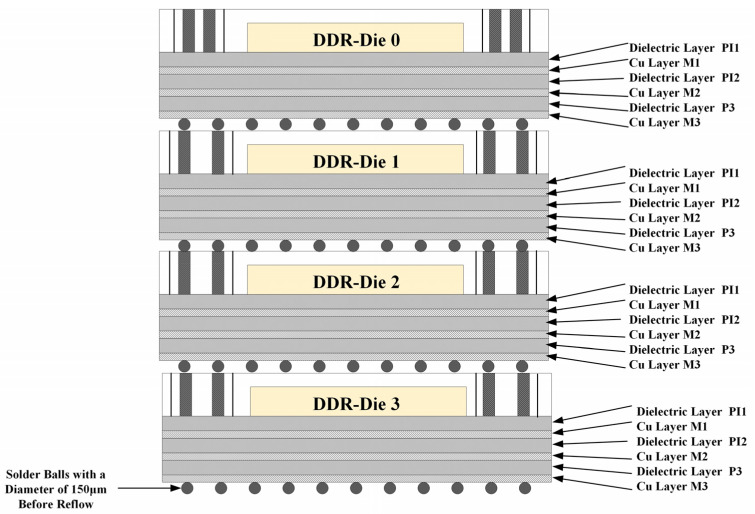
DDR memory micro-module integration process solution.

Due to the four-layer three-dimensional stacking configuration, the three lower chips (Die1–Die3) require routing on both the top and bottom surfaces. A structural diagram of the stacked DDR3 micro-module is shown in Figure 8. The specific parameters are as follows: the TSV diameter is 30 μm, height is 200 μm (aspect ratio of 1:6.7), pitch is 200 μm, and the number of TSVs per single-layer die is 360. Vertical signal continuity is achieved through high-density bump soldering. This double-sided redistribution structure poses significant process challenges. Consequently, this research focused on overcoming key technical hurdles in high-precision chip reconstruction processes. By optimizing critical steps, the electrical performance of the redistribution layer is ensured, ultimately meeting the stringent signal integrity requirements for DDR3 micro-module signal testing.

For the processing route adopted in this work, the warpage control of the single-layer fan-out chip is a critical factor affecting the process feasibility and final yield of the four-layer stacked DDR3 micro-module [23]. Studies indicate that wafer warpage not only induces processing defects such as micro-bump connection failure, uneven underfill filling, and chip cracking by disrupting the coplanarity of the bonding interface, but it also leads to stress accumulation and transfer effects during multi-layer stacking, resulting in a nonlinear decline in both electrical test yield and thermal cycle reliability yield. Therefore, the systematic implementation of warpage simulation, process parameter design of experiments (DOE) optimization, and model calibration are essential prerequisites for achieving the high-yield, high-reliability engineering realization of multi-layer stacked micro-modules.

To address the above issues, this paper proposed a simulation-based process optimization method aimed at mitigating wafer warpage caused by the mismatch of the coefficients of thermal expansion during wafer-level compression molding. To identify the key process parameters, the Taguchi experimental design method was employed to conduct warpage simulation analysis and process verification. Firstly, chip size, package extension ratio, chip thickness, and molding compound thickness were selected as key influencing factors, with three typical levels assigned to each factor to construct an orthogonal experimental matrix. The DOE parameters for the wafer warpage simulation are presented in Table 2. Based on this DOE scheme, warpage simulations were performed to determine the dominant factors affecting warpage. Subsequently, actual process verification was carried out using test chips, and the measured warpage data were compared with the simulation results to calibrate and optimize the warpage simulation model. Ultimately, a set of process parameters capable of effectively controlling wafer warpage was established, providing a quantifiable process window for the engineering manufacturing of the multi-layer stacked micro-module.

**Figure 9:**
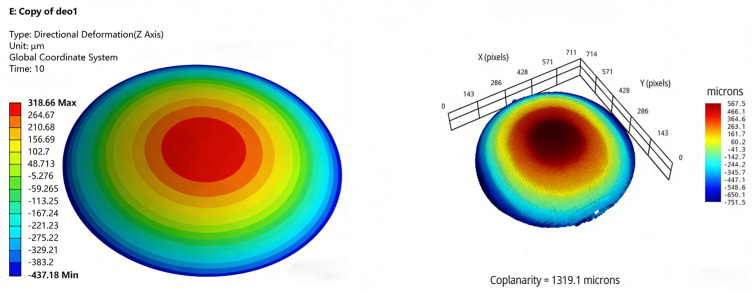
Warping simulation cloud map and measured cloud map (Test No. 1).

**Figure 10:**
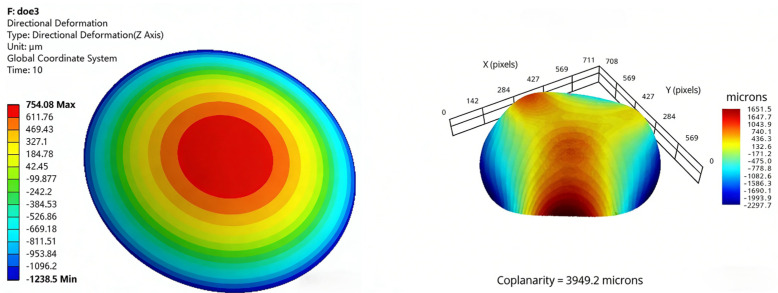
Warping simulation cloud map and measured cloud map (Test No. 3).

**Figure 11:**
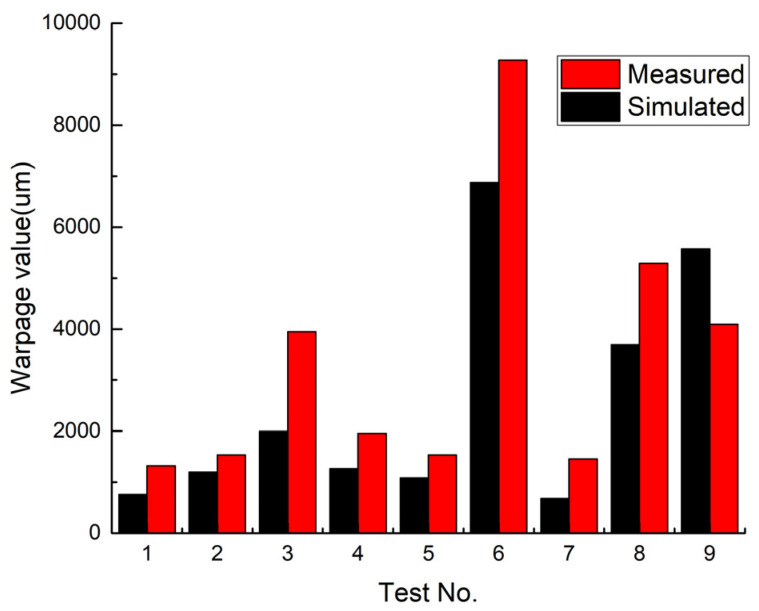
Comparison of warpage simulation and measured results.

The simulation conditions were initially set to a stress-free state at 125 °C, and the warpage resulting from the coefficient of thermal expansion (CTE) mismatch between the chip and the molding compound was analyzed after cooling from 125 °C to 25 °C. The simulation software and settings are provided in Table 3, while the material properties and related parameters are listed in Table 4. Meanwhile, dummy chips were used to fabricate samples under various process conditions, and warpage values were measured using the shadow moiré method. Taking Test No. 1 and Test No. 3 as examples, the simulation and measurement cloud maps are presented in Figure 9 and Figure 10. The comparative results for all nine test cases are shown in Figure 11.

After comparing the simulation and experimental results of warpage for plastic-encapsulated wafers, it was observed that with the exception of Test No. 9, the overall trend in the remaining experiments was generally consistent with the simulation results. Based on the provided simulation and experimental data, the coefficient of determination (R^2^) was calculated to be approximately 0.714, and the root mean square error (RMSE) was approximately 1334 μm. Regarding the significant discrepancies between the measured and simulated warpage values observed in Test Nos. 3, 6, 8, and 9, the reasons for them are mainly attributed to the following three factors: (1) the high flexibility of the process samples, which leads to multidirectional warpage deformation under their own weight; (2) the influence of the sample placement method, where the wafer edge is uniformly supported, while the central region (chip area) relies solely on self-weight, resulting in poor stability and fluctuations in warpage measurements; and (3) the reflective nature of the chip material surface, which interferes with the measurement accuracy of the detection equipment, thereby introducing significant calculation errors.

To further improve the consistency between the simulation and experimental results, future work can be optimized in the following aspects: (1) adopting a viscoelastic constitutive model to describe the time–temperature dependence of the molding compound and simulating the entire curing and cooling process; (2) obtaining the temperature-dependent modulus and CTE curves of the same batch of epoxy molding compound (EMC) through independent material testing, followed by the inverse calibration of uncertain parameters; and (3) optimizing the experimental measurement methods, such as employing multi-point laser scanning and surface matte treatment, to reduce reflection interference. It is anticipated that these improvements will increase the coefficient of determination to above 0.85 and reduce the RMSE to within 800, thereby providing a more reliable methodological foundation for high-precision warpage prediction in similar structures.

**Figure 12:**
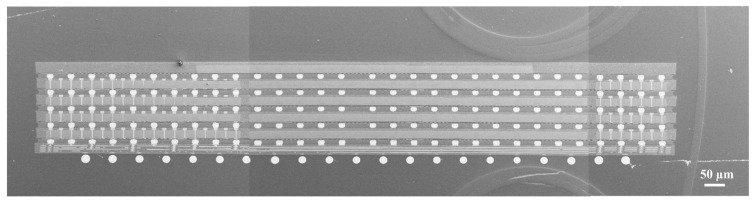
A SEM image of the DDR3 micro-module.

Based on the identification of key process parameters—including chip thickness, fan-out area, and molding compound thickness—a DOE approach was further employed to optimize the wafer-level compression molding process. The optimized parameters encompassed pre-baking, molding, post-curing, high-temperature debonding, molding force, and calendering speed. Through process validation, the optimal parameter set is as summarized in Table 5. The experimental results confirm that under these conditions, warpage in the single-layer wafer-level compression molding of the DDR3 micro-module is controlled within 8 μm. A scanning electron microscope (SEM) image of the DDR3 micro-module is presented in Figure 12.

This SEM image visually demonstrates the alignment accuracy and interconnection morphology achieved by the four-layer stacking process, confirming the feasibility of the proposed wafer-level fan-out and 3D stacking scheme. Additionally, it clearly presents the interface conditions and critical dimensions of each functional layer, providing a direct physical reference for subsequent warpage analysis, signal testing, and reliability evaluation.

## 4. Test Design and Implementation for Multi-Layer Stacked High-Capacity DDR3 Micro-Module

A multi-level test plan was designed for the DDR3 micro-module, covering wafer-level testing, post-redistribution testing, and multi-layer stacking testing. This comprehensive approach aims to evaluate whether the electrical parameters and functional performance of the modules meet the specified requirements [24].

The electrical parameter testing method follows the bare die manual. For functional testing, the DDR3 interface is initialized and configured, followed by full-address read and write operations at a data access speed of ≥800 Mbps. The test procedure consists of three steps: first, writing 0 × 55 AA to all addresses, then reading the data and verifying consistency; second, writing 0 × AA55 to all addresses, followed by read and comparison; and finally, writing the address value to all addresses, reading the data, and performing a comparison.

After functional testing and electrical parameter validation, the DDR3 micro-module demonstrated the ability to transmit data normally at a rate of 1600 Mbps. The tests were conducted under three temperature conditions: −55 °C, 25 °C, and 125 °C.

## 5. Conclusions

This paper presents a schematic design methodology and system-level integrated modeling scheme for a four-layer TSV vertically stacked DDR3 micro-module based on wafer-level packaging. By employing multi-chip heterogeneous fan-out technology and a high-precision TSV three-dimensional stacking process, the DDR3 micro-module was successfully fabricated. The entire process flow is built upon an independent mid-end process platform and does not rely on advanced process nodes or restricted equipment, forming a differentiated development path compared to HBM solutions that depend on advanced overseas processes, thereby offering significant advantages in terms of technological autonomy and supply chain security.

On this basis, a multi-level test system covering all internal nodes of the micro-module was constructed to conduct comprehensive functional and performance validation. The test results demonstrate that the fabricated micro-module meets all predefined design specifications. This work establishes a complete technical chain encompassing design methodology, process implementation, and test verification, providing referable design guidelines and a systematic process validation basis for the engineering development of similar three-dimensional stacked memory micro-modules.

## Figures and Tables

**Table 1:** Signal integrity simulation settings.

Item	Specific Settings
Simulation Software	Ansys SIwave and Circuit (version 17.2)
Models	The chip IBIS model, substrate, and micro-module layout design files
Simulation Criteria	JEDEC DDR3 standard [20] (AC150/DC100)@1600 Mbps

**Table 2:** DOE analysis for wafer warping simulation.

Test No.	Factor			
	Bare Chip Size (mm)	Chip Thickness (μm)	Fan-Out Ratio	Molding Thickness (μm)
1	1.67 × 1.51	600	2	150
2	1.67 × 1.51	400	4	200
3	1.67 × 1.51	100	6	300
4	10.31 × 8.06	600	4	300
5	10.31 × 8.06	400	6	150
6	10.31 × 8.06	100	2	200
7	5 × 5	600	6	200
8	5 × 5	400	2	300
9	5 × 5	100	4	150

**Table 3:** Simulation software and settings.

Item	Specific Settings
Simulation Software	ANSYS Mechanical (Version 2022R1)
Boundary Conditions	Thermal: Initial temperature of 125 °C, followed by natural cooling to 25 °C
	Mechanical: Free boundary condition applied at the bottom surface.

**Table 4:** Material Parameters.

Structure	Density (kg/m^3^)	Young’s Modulus (GPa)	Poisson’s Ratio	Coefficient of Thermal Expansion (10^−6^/°C)	Thermal Conductivity (W/M·°C)	Specific Heat Capacity (J/kg·°C)
Chip	2330	131	0.3	2.8	25 °C: 153 77 °C: 119 127 °C: 98.9	712
Epoxy Molding Compound	1660	14.21	0.3	13.8	2.1	1672

**Table 5:** Optimization methods for wafer-level compression molding processes.

Optimization Item	Optimization Method	Specific Methods
Pre-baking	130 °C	Pre-baking of the bonding film aims to increase the bonding force between the bonding film and the chip, without affecting warpage after plastic packaging
Plastic Encapsulation	125 °C	Set the baking time to 8 min to 12 min, conducting baking experiments in 1 min increments
Post-curing	150 °C	Set the baking time to 50 min to 70 min, conducting baking experiments in 10 min increments
High-temperature Degumming	180 °C~210 °C	Set the degassing time between 40 and 80 s, conducting degassing experiments in 10 s increments
Forming Force	220 kN	Set the molding force between 150 and 270 kN, conducting molding force experiments in 20 kN increments
Rolling Speed	10 mm/s	Set the calendering speed between 5 and 12 mm/s, conducting calendering speed experiments in 2 mm/s increments

## Data Availability

The data presented in this study are available on request from the corresponding author. The data are not publicly available due to confidentiality request.
